# Carbon felt modified with bismuth and asphalt-derived carbon as a high-performance electrode for vanadium redox flow batteries

**DOI:** 10.1371/journal.pone.0324878

**Published:** 2025-05-28

**Authors:** ZHI-CHEN ZHOU

**Affiliations:** College of Energy, Soochow University, Suzhou, China; Chulalongkorn University, THAILAND

## Abstract

Vanadium redox flow batteries (VRFBs) are among the most promising large-scale energy storage systems, owing to high efficiency, scalability, and long cycle life. However, their widespread adoption is often hindered by sluggish electrode reaction kinetics, particularly at the anode. This investigation aimed to address these limitations by introducing bismuth-doped carbon (Bi/C) nanoparticles synthesized from asphalt and bismuth onto thermally treated carbon felt (TCF) to prepare Bi and C co-deposited thermally treated carbon felt (Bi/C-TCF), leveraging the synergistic effects between the two components. The synthesis process involved spray drying followed by high-temperature calcination, resulting in a highly efficient electrocatalyst for the V^3+^/V^2+^ redox couple. Electrochemical testing revealed that the Bi/C-TCF electrode significantly outperformed the conventional TCF electrode, exhibiting reduced polarization during charge-discharge cycles and enhanced catalytic activity as evidenced by its superior reaction rate constants K^0^ (2.37 × 10^−2^ and 2.75 × 10^−2^ cm/s) compared to TCF (2.08 × 10^−2^ and 2.10 × 10^−2^ cm/s). In single-cell tests, the Bi/C-TCF electrode, used as the negative electrode, demonstrated superior voltage efficiency (VE) and energy efficiency (EE) across various current densities. It achieved a power density of up to 1054.3 mW/cm^2^, significantly outperforming TCF’s 825.9 mW/cm^2^. After 1000 cycles, the VE and EE remained stable at 86.2% and 85.0%, respectively, whereas the TCF cell saw a rapid decline in VE and EE to below 70% after just 515 cycles. These findings highlight the potential of Bi/C nanoparticles as a scalable and cost-effective solution for enhancing the performance and durability of VRFBs, leveraging low-cost raw materials such as asphalt.

## 1. Introduction

With the aggravation of greenhouse effect and escalating environmental pollution, the global demand for green energy is witnessing a marked increase [1–3]. This trend compels renewable energy factories to expand operations in a manner that is not only more efficient but also aligned with environmental sustainability, necessitating the exploration of alternatives to conventional fossil fuels. However, while striving to satisfy the peak demands of the power grid, these renewable energy sources must concurrently address the challenges posed by the intermittent nature of energy supply, which is inherently dependent on local geographical factors and stochastic weather conditions [2–8]. The absence of an integrated energy storage system constitutes one of the principal impediments to the widespread adoption of renewable energy, a limitation that places it at a comparative disadvantage relative to traditional power generation methods.

Among various energy storage technologies, the redox flow batteries (RFBs) have emerged as promising candidates for large-scale energy storage [9–15]. Unlike other battery systems, RFBs are characterized by their distinctive architecture, wherein the electrolyte and active materials are stored in separate external reservoirs, with the electrolyte itself serving as the energy storage medium. The electrolyte is continuously circulated to the electrode surface through a peristaltic pump and is separated by an ion-exchange membrane [16]. This design ensures that the electrodes only provide a site for redox reactions without participating in the reactions themselves, inherently endowing RFBs with a prolonged operational lifespan. Moreover, the intrinsic ability of RFBs to decouple energy from the power is a significant advantage, providing considerable flexibility in the design process to tailor the battery for specific energy storage applications. This can be achieved by manipulating variables such as the concentration of active species [17] and the number of electrons transferred [18]. RFBs can be customized to meet a wide range of energy storage demands. The rapid response time, low self-discharge rate, and resilience to overcharging and deep discharging further enhance the viability of RFBs as an effective energy storage technology.

VRFBs exemplify the general advantages of RFBs while offering distinct benefits due to the utilization of identical vanadium species, VO_2_^+^/VO^2+^ for the positive half-cell and V^3+^/V^2+^ for the negative half-cell, thus eliminating the possibility of cross-contamination between electrolytes. Furthermore, the absence of a separation step in the recycling process contributes to a reduction in overall battery recycling costs [19–22]. Currently, carbon felt (CF) has been a crucial material for electrodes of VRFBs, which possesses low manufacturing cost, favorable electrical conductivity, and high mechanical strength. However, CF materials are characterized by poor electrochemical activity and suboptimal wettability, which impede efficient electrolyte penetration. Under prolonged exposure to highly acidic electrolytes, the surface morphology of carbon fibers undergoes significant alterations as the reaction progresses, severely limiting the performance of VRFBs [23–25]. Consequently, substantial research attention has been directed towards the modification of CF materials to enhance its electrochemical properties, mainly including: (1) surface etching techniques, which introduce oxygen-containing functional groups onto the CF materials through thermal or acid treatments, increase specific surface area, and improve wettability [26–33]; (2) doping with heteroatoms such as phosphorus (P) [34,35], nitrogen (N) [36–38], and sulfur (S) [39,40], which can introduce additional redox-active sites; (3) incorporating of electrocatalysts, including various metals and metal oxides, to accelerate electron transfer kinetics and mitigate polarization effects [41].

Given that the electrolyte in VRFBs is typically highly acidic, with a substantial concentration of hydrogen ions, the hydrogen evolution reaction (HER) may emerge as a side reaction at the negative electrode, significantly compromising the overall energy efficiency of the system. Consequently, the modification of CF materials to reduce polarization can substantially enhance its energy efficiency and power density. Previous research has demonstrated that bismuth (Bi), a low-toxicity, high-density, and cost-effective metal, exhibits a strong catalytic effect on the V^3+^/V^2+^ redox couple [7,12]. According to the mechanism proposed by Suarez et al. [7], the reduction of H^+^ occurred preferentially on the surface of deposited bismuth rather than on the carbon substrate, thereby significantly reducing the efficiency losses associated with the HER and the degradation of oxygen-containing functional groups. Furthermore, Bi can improve the efficiency of the oxidation of V^2+^ to V^3+^, which has been established as the rate-determining step [42].

The modification of novel carbon-based catalysts is also a significant area of research. Carbon-based materials, which share identical elemental similarities with the carbon felt substrate, inherently confer advantages such as an increased specific surface area and the proliferation of active sites. Notable examples include graphene [43,44], carbon nanotubes (CNT) [45], graphene oxide (rGO) [46], and carbon nanoparticles [47]. These materials exhibit exceptional stability and electrical conductivity, offering the potential to further optimize the EE of VRFBs. However, the modification processes for these materials are often complex and costly, and they frequently failed to address the intrinsic hydrophobicity of the electrode surface, which remained a limiting factor in catalytic performance. From a compositional perspective, asphalt is a hydrocarbon with high carbon content, adjustable morphology, and crystallinity, which presents a promising, low-cost carbon precursor [48–51].

Building on the previous discussion, we propose a straightforward spray-coating method to incorporate Bi/C nanospheres onto TCF, creating Bi/C-TCF electrodes. Using TCF, Bi-TCF, and C-TCF as control groups, we evaluated the performance of these electrodes in VRFBs to explore the synergistic effects of Bi and C co-deposition on the negative electrode reactions. The findings indicated that the Bi/C-TCF electrode had a high specific surface area and enhanced hydrophilicity, with the water contact angle decreasing from 138° for TCF to 58°. As a result, in half-cell tests, the charge transfer resistance ($R_{ct}$) of Bi/C-TCF was 1.0 Ω, significantly lower than that of TCF at 3.3 Ω. Tafel plots further confirmed the reduced polarization of Bi/C-TCF. In full-cell tests, VRFBs employing Bi/C-TCF anodes exhibited enhanced VE and EE, outperforming TCF anodes by 5.1–8.3% across high current densities (100–300 mA/cm^2^). Furthermore, the Bi/C-TCF single cell exhibited excellent stability over 1000 charge-discharge cycles and achieved a power density of 1054.3 mW/cm^2^, significantly surpassing that of TCF single cells, indicating reliable scalability. This approach not only enhanced the value of waste asphalt but also improved VRFB performance, offering valuable insights for commercial applications in large-scale energy storage.

## 2. Materials and methods

### 2.1. Chemicals and materials

The waste asphalt was provided by Dongying Guangfa Chemical Co., Ltd. and conformed to the national standard No. 10 for paving asphalt. All chemicals were purchased from Aladdin (analytical reagent grade) and used without further purification. The carbon felt was supplied by Liaoning Jingu Co., Ltd.; the separator was Nafion 212 (DuPont, USA); and the electrolyte was provided by Hunan YinFeng Co., Ltd.

### 2.2. Preparation of carbon felt modified electrodes

To prepare the modified CF electrodes, the pristine CF underwent a thorough cleaning process to remove dust and other impurities. Initially, the CF was immersed in ethanol and subjected to ultrasonic cleaning for 30 min. Subsequently, the CF was ultrasonically cleaned in distilled water for 15 min. This cleaning cycle was repeated three times to ensure the complete removal of the contaminants. Following this, the cleaned CF was activated in a muffle furnace at 400°C for 6 h with a controlled heating rate of 10°C/min, resulting in TCF.

The asphalt-based carbon precursor was prepared by dissolving 50 g raw asphalt in 500 mL dichloromethane. The mixture was ultrasonicated for 30 min, forming a homogeneous black suspension. After centrifugation to remove insoluble impurities, the supernatant was collected, and the solvent was evaporated at 120°C to obtain pretreated asphalt.

The pretreated asphalt was then subjected to pyrolysis in a tube furnace at 800°C for 12 h and cooled naturally to room temperature, resulting in the formation of carbon microspheres. The carbon microsphere powder was mixed with bismuth powder at a 3:2 mass ratio and ball-milled using zirconia grinding balls of varying diameters with a ball-to-powder ratio of 6:1 (w/w). Ethanol was employed as the wet-milling medium, and the process was conducted at 400 rpm for 12 h in a cyclic mode (30 min of forward rotation, 5 min of rest, 30 min of reverse rotation). The resulting Bi/C composite exhibited particle diameters ranging from 50 to 100 nm.

To prepare the modified electrodes, Bi/C powder was dispersed in a mixture of Nafion and anhydrous ethanol (at a mass/volume ratio of 1 mg Bi/C: 20 μL Nafion: 1 mL anhydrous ethanol). For TCFs of different sizes, the mass ratio of Bi/C particles to TCF was controlled at 1:50. During spraying, a pneumatic spray gun was employed to atomize the suspension with a fixed gas flow rate, where the atomization size and nozzle-to-TCF distance were optimized to achieve a wetting diameter of ~0.5 mm at a fixed distance of 5 cm. Additionally, a constant-speed manual movement along an S-shaped path was adopted, with the spray trajectory extending 1–2 cm beyond the substrate edges to compensate for prolonged dwell time at inflection points. Furthermore, a dual-sided alternating spraying strategy ensured that the suspension infiltrated the carbon fiber surface and internal pores in thin, sequential layers, achieving complete wetting. The resulting Bi/C-TCF electrodes were then dried at 80°C.

As controls, two additional samples were fabricated: (1) C-TCF, prepared by dispersing and spraying asphalt-derived carbon onto TCF without bismuth incorporation, and (2) Bi-TCF, synthesized by dispersing and spraying pure bismuth powder (without asphalt-based carbon) onto TCF. Both control samples underwent identical spraying and drying procedures as the Bi/C-TCF electrode, ensuring consistency in the fabrication process.

### 2.3. Physicochemical characterization

The surface morphology of the TCF and Bi/C-TCF was characterized by scanning electron microscopy (SEM; JEOL JSM-7900F) at an acceleration voltage of 10 kV. To analyze the details of the crystallographic structure, High-resolution transmission electron microscopy (HRTEM; FEI Talos F200X) was performed at 200 kV to observe the Bi/C particles. The surface element distribution was analyzed using energy-dispersive spectroscopy (EDS; Bruker Quantax 75). X-ray photoelectron spectroscopy (XPS; Thermo Fisher Scientific K-Alpha) was conducted using a monochromatic Al K_α_ source (1486.6 eV) to investigate the surface chemical composition and bonding states. The binding energies were calibrated with reference to the C 1s peak at 284.8 eV. The crystal structure was examined by X-ray diffraction (XRD; Rigaku Ultima IV) with Cu K_α_ radiation (λ = 1.5406 Å), and phase composition was identified by matching the diffraction patterns with reference data from the ICDD database. Contact angle measurements were performed using a sessile drop method to assess the hydrophilicity of the samples.

### 2.4. Electrochemical characterization

Cyclic voltammetry (CV), electrochemical impedance spectroscopy (EIS), and Tafel polarization tests were performed using a traditional three-electrode setup at room temperature on a Biologic VMP multi-channel potentiostat/galvanostat. The working electrodes (WE) were TCF, C-TCF, Bi-TCF or Bi/C-TCF with an effective area of 1 × 1 cm^2^. The reference electrode (RE) was a standard Hg/Hg_2_SO_4_ electrode (0.656V vs. SHE, 25°C), while a platinum wire served as the counter electrode (CE). All electrochemical tests were conducted in an electrolyte containing 0.1 M VOSO_4 _+ 1.0 M H_2_SO_4_. The electrolyte was purged with high-purity nitrogen gas to remove dissolved oxygen and prevent any oxidative effects of V^3+^/V^2+^on the electrochemical responses. This procedure ensured the accuracy and reliability of the measurements, providing insights into the electron transfer and diffusion characteristics of the studied system.

The CV measurements were conducted by sweeping the potential from 0 V to −0.9 V (initial scan direction: cathodic) at a scan rate of 10 mV/s for 3 cycles to characterize the V^3+^/V^2+^ redox couple. For EIS, the AC impedance spectra were captured over a frequency range from 100 kHz to 10 mHz with an AC amplitude of 10 mV. Tafel curves were obtained by sweeping the potential from the open-circuit potential (OCP) to +250 mV (anodic) and −250 mV (cathodic) at a scan rate of 1 mV/s.

### 2.5. Cell tests

In the test of single-cell, the anolyte contained 1.5 M V^3+^ in 3.0 M H_2_SO_4_, while the catholyte comprised 1.5 M VO^2+^ in 3.0 M H_2_SO_4_. The volume of each electrolyte solution was maintained at 70 mL. The negative electrode was constructed from TCF or Bi/C-TCF, and the positive electrode utilized TCF. The single cell had an effective surface area of 4 × 4 cm^2^. A Nafion 212 membrane served as the separator, and the electrolyte flow rate was regulated by a peristaltic pump set to 65 mL/min. The VRFB performance was evaluated using a CT-4008T battery test system (Neware Co., Ltd.), with operational voltage boundaries set between 0.70 V and 1.75 V.

To evaluate the rate performance, cyclic charge-discharge tests were conducted at various constant current densities (50, 100, 150, 200, 250, 300, and back to 50 mA/cm^2^), with five cycles performed at each current density. Following the rate tests, additional charge-discharge cycles were conducted at current densities of 50, 100, 150, 200, 250, and 300 mA/cm^2^ to evaluate the long-term stability and high-current-density tolerance of the materials. To further investigate the electrochemical kinetics and activity of the electrode reactions, the galvanostatic intermittent titration technique (GITT) was employed. At a current density of 40 mA/cm^2^, the VRFB single cell underwent alternating 4-minute charging/discharging phases, each followed by a 4-minute open-circuit relaxation. For polarization and power density tests, a graphite plate cell with an active area of 2.5 × 2.5 cm^2^ was used at a 100% state of charge (SOC). The polarization test was initiated with a starting current of 260 mA, which was incrementally increased at a rate of 10 mA/s. The test was terminated when the current reached 10 A or when the cell voltage dropped to 0V. The power density metrics were obtained from these polarization tests to determine the maximum power output and overall efficiency of the cell. All the experimental measurements were conducted at 25 ± 2°C.

These comprehensive assessments provide an in-depth evaluation of the performance, stability, and efficiency of the VRFB single cell, offering critical insights into its potential for practical energy-storage applications.

## 3. Results and discussion

**Fig 1:**
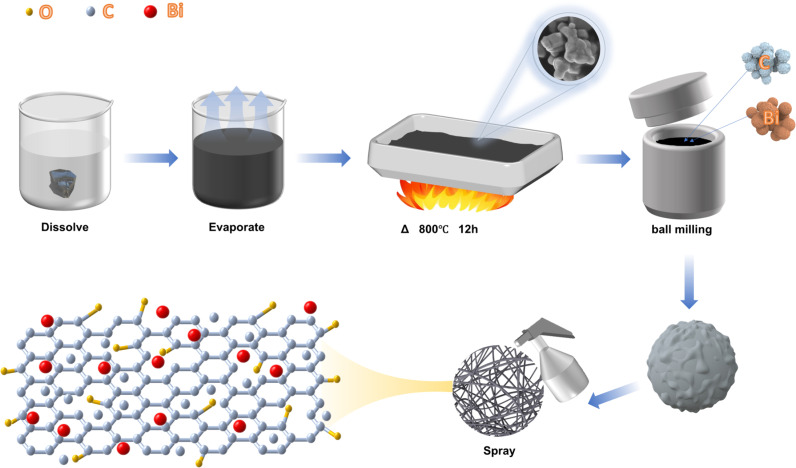
Preparation of Bi/C-TCF.

Fig 1 illustrates the meticulous preparation process of Bi/C-TCF. Within the experimental framework, the ball-milling method played a crucial role in controlling particle size, which led to the formation of uniformly dispersed nanospheres. The reduction in particle size significantly increased the specific surface area, thereby exposing a greater number of active sites [52]. Additionally, high-temperature calcination was employed to introduce oxygen-containing functional groups on the particle surfaces. This process further increased the density of active sites, facilitated nanoscale regulation of particle size, and promoted the homogeneity of the Bi/C composite. The introduction of these functional groups during calcination was critical because they enhanced the electrochemical reactivity of the material. This synergy between particle size reduction and surface functionalization was pivotal for optimizing the catalytic properties of Bi/C-TCF electrodes.

**Fig 2:**
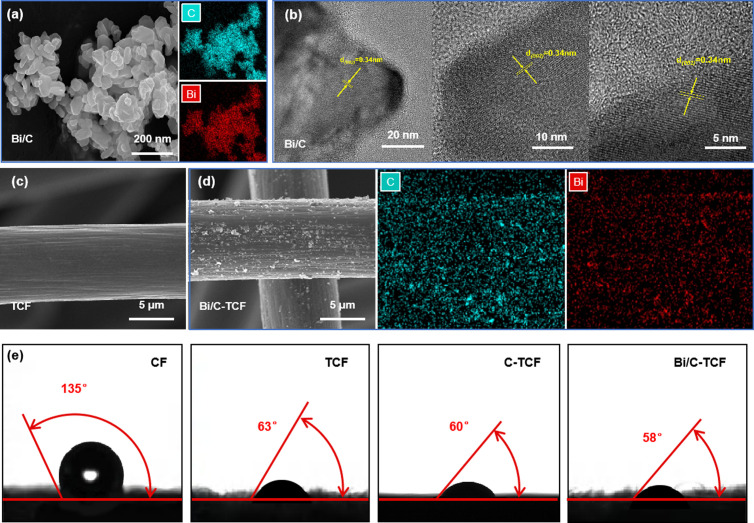
(a) SEM image and corresponding EDS mapping of Bi/C nanoparticles. (b) HRTEM image of Bi/C nanoparticles. (c) SEM image of untreated TCF. (d) SEM image and EDS mapping of Bi/C-TCF. (e) Cross-sectional views of water droplet contact angles on CF, TCF, C-TCF, and Bi/C-TCF surfaces.

SEM images revealed the morphology of Bi/C particles after ball milling and the changes in material surface morphology before and after treatment. Fig 2a shows that Bi/C particles were successfully controlled at the nanoscale through ball milling, resulting in particles that predominantly exhibited a spindle shape and were closely packed together. The image also revealed numerous pores and gaps between the particles, which increased the surface area and introduced defects that could serve as active sites. HRTEM analysis further revealed the crystalline nature of the Bi/C particles, where distinct lattice fringes with an interplanar spacing of approximately 0.34 nm were observed, corresponding to the (002) plane of graphitic carbon, as shown in Fig 2b. This confirmed the presence of a well-ordered graphitic structure within the composite, which is critical for facilitating efficient electron transfer and enhancing electrochemical activity. Fig 2c illustrates the microstructure of untreated TCF, where the surface is characterized by smooth vertical stripes. In contrast, Fig 2d depicts the Bi/C-TCF surface after treatment, revealing substantial deposition of Bi/C particles. These particles are primarily localized within the folds of the TCF, generating numerous defect sites that could significantly enhance the electrochemical activity of the material. EDS mapping clearly demonstrated the uniform distribution of Bi and C elements across both the Bi/C particles and the Bi/C-TCF composite, confirming the successful incorporation of Bi/C into the TCF framework.

Contact angle experiments demonstrated improved electrode wetting with the electrolyte under various treatment methods. The inherent hydrophobic nature of graphite materials frequently impeded effective electrolyte penetration on the surface of the VRFB electrodes. As shown in Fig 2e, for the pristine CF, the contact angle was 135°, which indicated poor hydrophilicity. However, after heat treatment, the contact angle of TCF decreased significantly to 63°, primarily due to the increased number of oxygen-containing functional groups on the CF surface. Notably, C-TCF exhibited a contact angle of 60°, slightly lower than TCF but higher than Bi/C-TCF (58°). This suggests that Bi further enhances hydrophilicity beyond the effect of carbon alone. The improvement in Bi/C-TCF is likely due to the combined effects of oxygen groups and Bi’s hydrophilic nature.

**Fig 3:**
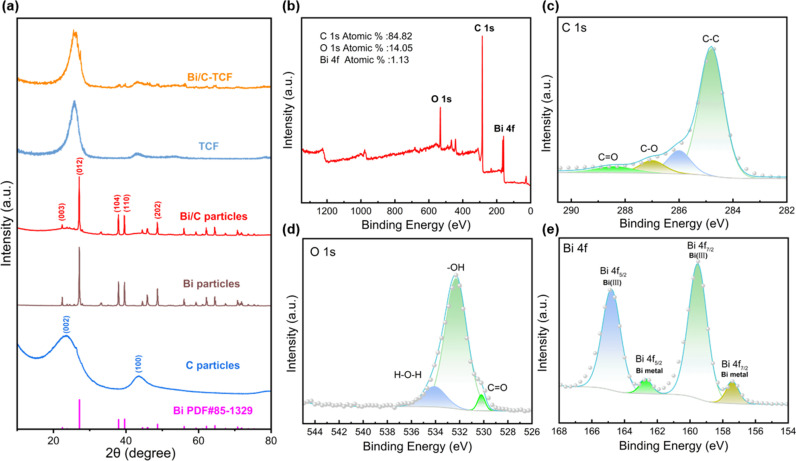
(a) XRD patterns of C particles, Bi particles, Bi/C particles, TCF, Bi/C-TCF, and standard PDF card of Bi. (b) XPS survey scan of Bi/C-TCF composite. High-resolution XPS spectra of (c) C 1s region, (d) O 1s region, and (e) Bi 4f region.

XRD was used to analyze the structures of C particles, Bi particles, Bi/C particles, TCF, and Bi/C-TCF, as shown in Fig 3a. For the C particles, two broad diffraction peaks at 23.7° and 43.5° were observed, corresponding to the (002) and (100) planes of the graphite lattice. The broadened (002) peak indicated a low degree of graphitization and abundant structural defects in the carbon matrix, which could enhance interfacial interaction with Bi species during composite formation. Both Bi particles and Bi/C particles exhibited sharp diffraction peaks at 27.2° (012), 37.9° (104), and 39.6° (110), corresponding to the rhombohedral metallic Bi phase (PDF#85–1329), along with a weak peak at 33.2° attributed to partial surface oxidation, which is consistent with previously reported patterns [53]. The Bi/C particles exhibited a superimposed C and Bi diffraction profiles, demonstrating effective integration of both components while maintaining the structural integrity of carbon and preserving the crystallinity of the metallic Bi regions.

For TCF and Bi/C-TCF, both samples demonstrated two prominent peaks at 25.6° and 43.7°. However, the diffraction peaks of Bi/C-TCF exhibited notably reduced intensity and increased breadth compared to those of TCF. According to the Scherrer Equation, an increase in the observed Full Width at Half Maximum (FWHM) indicated that the crystallite size on the Bi/C-TCF was smaller [54]. Compared to the standard Bi PDF card, confirming metallic Bi incorporation. This modification introduced lattice defects into the electrode material, which might function as active centers[55], thereby enhancing the electrochemical reactivity and overall performance.

The XPS characterization systematically revealed the surface chemical states and elemental composition of the Bi/C-TCF composite. As shown in the survey spectrum (Fig 3b), prominent C 1s, O 1s, and Bi 4f peaks were detected, with atomic percentages of 84.82% (C), 14.05% (O), and 1.13% (Bi), respectively. The high-resolution C 1s spectrum (Fig 3c) was deconvoluted into four components: graphitic carbon (C-C, 284.8 eV, 73.78%), defective carbon (286.0 eV, 12.74%), C-OH (287.1 eV, 7.60%), and -COOH (288.4 eV, 5.89%). These oxygen-containing functional groups not only improved the electrode’s hydrophilicity but also provided additional active sites for vanadium redox reactions. Specifically, the -OH groups serve as versatile precursors for surface modification [56], while the -COOH groups facilitate the adsorption of positively charged ions [57]. The O 1s spectrum (Fig 3d) resolved three distinct peaks at 530.3 eV (C = O, 3.54%), 532.3 eV (-OH, 87.12%), and 534.1 eV (adsorbed H_2_O, 9.33%), corroborating the surface functionalization observed in the C 1s analysis [58]. The Bi 4f spectrum (Fig 3e) exhibited characteristic spin-orbit splitting with Bi 4f_7/2_ and Bi 4f_5/2_ components, each split into two sub-peaks indicating mixed valence states. The dominant peaks at 159.5 eV (Bi 4f_7/2_) and 164.9 eV (Bi 4f_5/2_) correspond to Bi^3+^ species, whereas the lower binding energy peaks at 157.2 eV (Bi 4f_7/2_) and 162.5 eV (Bi 4f_5/2_) suggest the coexistence of reduced Bi^0^ states. This mixed-valence configuration likely enhances electrochemical activity through synergistic electron transfer effects.

**Fig 4:**
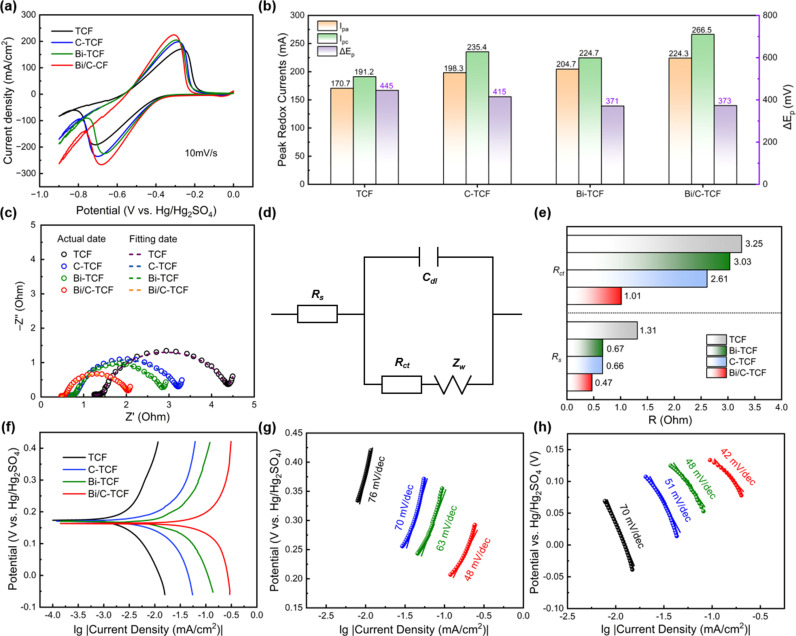
(a) CV curves of TCF, C-TCF, Bi-TCF, and Bi/C-TCF recorded at a scan rate of 10 mV/s. (b) Statistical table of peak currents ($I_{pa}$ and $I_{pc}$) and peak potential separations ($\Delta E_{p}$) derived from the CV curves. (c) Nyquist plots of the EIS of TCF, C-TCF, Bi-TCF, and Bi/C-TCF. (d) The equivalent circuit of the EIS. (e) $R_{s}$ and $R_{ct}$ values of TCF, C-TCF, Bi-TCF, and Bi/C-TCF. (f) Tafel polarization curves of TCF, C-TCF, Bi-TCF, and Bi/C-TCF. (g)-(h) Tafel slope curves.

CV tests were conducted in a three-electrode system to evaluate the electrochemical performance of TCF, C-TCF, Bi-TCF and Bi/C-TCF as negative electrodes for the V^3+^/V^2+^ redox reaction. The electrocatalytic activity was evaluated by measuring the peak currents ($I_{pa}$and$I_{pc}$) and the peak potential separation ($\Delta E_{p}$=$E_{pa}$−$E_{pc}$). As shown in Fig 4a, the Bi/C-TCF electrode exhibited significantly enhanced $I_{pc}$ values and $I_{pa}$ values, indicating superior electronic/ionic conductivity. Furthermore, Bi/C-TCF demonstrated the smallest $\Delta E_{p}$(373 mV), which was 53 mV and 72 mV lower than those of C-TCF (426 mV) and TCF (445 mV), respectively. Notably, although Bi-TCF showed a slightly smaller $\Delta E_{p}$(371 mV) compared to Bi/C-TCF, its peak current densities were significantly lower, resulting in an overall inferior performance to Bi/C-TCF. This smaller $\Delta E_{p}$ indicated a higher reversibility and lower polarization effect for the V^3+^/V^2+^ redox couple when Bi/C-TCF was employed as the negative electrode. Statistical representations of the peak currents and peak potential separation for the four electrodes were presented in Fig 4b.

As the scan rate increased, both the $I_{pa}$ and $I_{pc}$ of TCF and Bi/C-TCF electrodes exhibited proportional enhancement (S1 Fig a, b), confirming the diffusion-controlled nature of these redox processes governed by mass transfer of redox-active species. Notably, the Bi/C-TCF electrode demonstrated significantly larger $I_{p}$ values and $\Delta E_{p}$ compared to TCF (S1, S2 Tables), indicative of superior electron transfer kinetics. Quantitative analysis revealed that the rate constants (K^0^) for Bi/C-TCF (2.37 × 10^−2^ cm/s and 2.75 × 10^−2^ cm/s) substantially exceeded those of TCF (2.08 × 10^−2^ cm/s and 2.10 × 10^−2^ cm/s) (S1 Fig c, d and S3 Table). These kinetic enhancements correlate with the observed superior mass transport capability of Bi/C-TCF, suggesting its porous architecture facilitates rapid adsorption of V^2+^/V^3+^ ions through synergistic effects between the conductive carbon framework and bismuth catalytic sites, while optimized surface chemistry promotes efficient charge transfer at the electrode-electrolyte interface [59,60]. Additional CV tests (S2 Fig) systematically validated the optimized synthesis parameters, confirming that the pyrolysis temperature of 800°C and a Bi/C mass ratio of 3:2 represent the most reasonable experimental conditions.

EIS provided further insight into the enhanced electrochemical performance of the Bi/C-TCF electrode. The Nyquist plots of TCF, C-TCF, Bi-TCF and Bi/C-TCF exhibited a high-frequency semicircular region and a low-frequency linear region, as shown in Fig 4c. The high-frequency semicircle was associated with the electron transfer process, while the low-frequency region, which approached a straight line, corresponded to the mass diffusion process. In the equivalent circuit shown in Fig 4d, the electrolyte resistance ($R_{s}$) represented the ohmic resistance of the electrode system, including the intrinsic resistance of the solution and the resistance encountered by electrons crossing the electrode/solution interface. This could be determined by reading the real axis at the high-frequency cut-off point of the Nyquist plot. The charge transfer resistance ($R_{ct}$) at the electrode-electrolyte interface reflected the kinetics of electron transfer during redox reactions and was represented by the diameter of the semicircle in the high-frequency region. $Z_{w}$ denotes the Warburg impedance associated with diffusion, while $C_{dl}$ signifies the double-layer capacitance. Fig 4e illustrated the $R_{ct}$ and $R_{s}$ values of the electrodes. $R_{ct}$ of the TCF electrode was 3.3 Ω, indicating limited electron transfer kinetics. Bi-TCF exhibited a $R_{ct}$ of 3.03 Ω, suggesting that bismuth alone provides minimal catalytic enhancement. Upon modification with asphalt-based carbon, the $R_{ct}$ of the C-TCF electrode decreased by 21.2%, reaching 2.6 Ω. Remarkably, the $R_{ct}$ of the Bi/C-TCF electrode was reduced to 1.0 Ω, which represented a 69.7% reduction from the original value of TCF. This substantial reduction in $R_{ct}$ suggested that the synergistic incorporation of carbon and bismuth facilitates interfacial electron transfer between vanadium ions and the electrode, endowing Bi/C-TCF with superior electrocatalytic activity compared to TCF, C-TCF, and Bi-TCF.

Tafel analysis provided critical insights into the electrocatalytic performance of the electrodes by correlating current density with overpotential. As shown in Fig 4f, the exchange current density ($j_{o}$) and corrosion potential ($E_{corr}$) were derived from extrapolating the linear regions of the Tafel plots. For Bi/C-TCF, the $j_{o}$ was higher than that of TCF, C-TCF, and Bi-TCF, indicating a more facile electrode reaction and enhanced electron transfer kinetics. The $E_{corr}$ values of TCF and C-TCF were nearly identical (174 mV and 170 mV), while Bi-TCF showed an $E_{corr}$ of 170 mV, similar to C-TCF. In contrast, Bi/C-TCF exhibited a more negative $E_{corr}$(163 mV), thermodynamically favoring the V^3+^/V^2+^ redox reaction at the negative electrode. Furthermore, the slope of the Tafel curve, which served as another indicator of reaction kinetics, is depicted in Fig 4g and Fig 4h. At a 100% SOC, Bi/C-TCF exhibited a smaller slope, which corresponded to a lower overpotential for the V^3+^/V^2+^ redox reaction at the same kinetic current density. The reduced overpotential underscores the enhanced catalytic activity of Bi/C-TCF for the V^3+^/V^2+^ redox reaction. This improvement arises from a synergistic interplay of thermodynamic favorability and kinetic superiority, demonstrating that the combined effect of bismuth and carbon is critical for achieving superior electrochemical performance, whereas individual components offer only marginal benefits.

**Fig 5:**
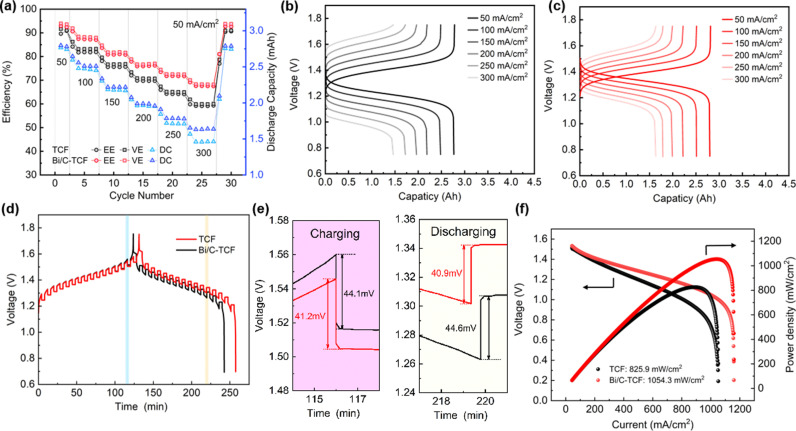
(a) Rate performance of VRFBs with TCF and Bi/C-TCF as anodes, respectively. (b)-(c) The capacity-voltage curves for VRFBs with TCF and Bi/C-TCF anodes at different current densities. (d)-(e) The GITT profiles of the VRFBs with TCF and Bi/C-TCF anodes at 40 mA/cm^2^, respectively. (f) The power density and polarization curves of VRFBs with TCF and Bi/C-TCF anodes, respectively.

To systematically evaluate the electrochemical activity and kinetic characteristics of Bi/C-TCF in single-cell configurations, rate performance analyses were performed on VRFBs employing TCF and Bi/C-TCF electrodes. As illustrated in Fig 5a, cell performance metrics including VE, EE and discharge capacity (DC) were measured across varying current densities. The Bi/C-TCF-based cells consistently outperformed TCF counterparts, maintaining superior VE and EE values at all tested densities. The DC analysis revealed performance differences between the electrodes, with Bi/C-TCF demonstrating slightly higher capacity than TCF below 200 mA/cm^2^. This disparity increased further above 250 mA/cm^2^, peaking at a 0.17 mAh advantage for Bi/C-TCF at 300 mA/cm^2^. At this current density, TCF exhibited significant performance degradation with VE and EE plummeting to 59.9% and 59.1%, respectively, while Bi/C-TCF retained stable efficiency at 68.3% VE and 67.6% EE. When the current density returned to 50 mA/cm^2^, the EE of Bi/C-TCF exhibited only a 0.01% decrease from its initial value, demonstrating its reversibility and high-current resilience. The capacity-voltage curves of VRFBs based on TCF and Bi/C-TCF at various current densities were shown in Fig 5b and 5c, respectively. As the current density increased, the voltage difference between the charging and discharging plateaus gradually widened, indicating an increase in polarization. Compared to TCF, Bi/C-TCF exhibited a lower charging plateau and a higher discharging plateau at high current densities. This suggested that Bi/C-TCF experienced less polarization during the charge-discharge process, making it more suitable for high-current charge-discharge applications. Figure 5d presents the results of the GITT for the VRFB. The results indicated that VRFB with Bi/C-TCF electrodes exhibited longer retention times during both charging and discharging, further suggesting lower polarization. The red and blue areas in Fig 5d represented a complete charge-discharge cycle, along with resting periods. As illustrated in Fig 5e, 5a detailed examination of the charging process revealed that the voltage drop for the Bi/C-TCF single cell was 41.2 mV at the onset of charging, which was less than the 44.1 mV drop observed for the TCF single cell. Similarly, during the discharge process, the Bi/C-TCF single cell exhibited a voltage rise of 40.9 mV at the onset, which was smaller than the 44.6 mV rise seen in the TCF single cell. In the power density and polarization curves presented in Fig 5f, the power density of the cell based on Bi/C-TCF was 1054.3 mW/cm^2^, while that of TCF was 825.9 mW/cm^2^. These test results above show that Bi/C-TCF exhibits outstanding catalytic performance for the V^3+^/V^2+^ redox couple, effectively reducing polarization in VRFBs, extending their lifespan, and significantly boosting power density.

**Fig 6:**
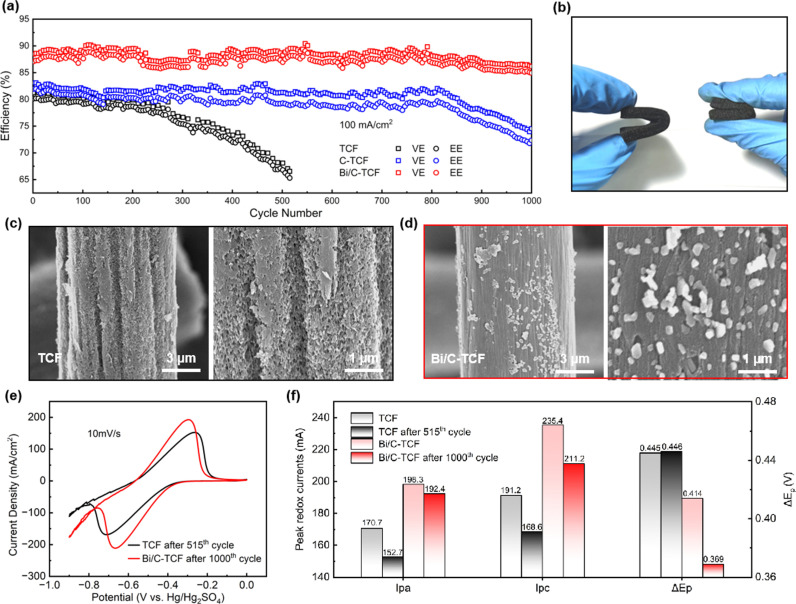
(a) The EE and VE of VRFB stacks with TCF, C-TCF and Bi/C-TCF as negative electrodes. (b) Photographs of the TCF electrode (left) and Bi/C-TCF electrode (right) before and after cycling. (c) SEM images of the TCF electrode after cycling. (d) SEM images of the Bi/C-TCF electrode after cycling. (e) CV curves of the electrodes after cycling. (f) Statistical table of $I_{pa}$_,_ $I_{pc}$ and $\Delta E_{p}$ from the CV curves.

To assess the long-term stability of the electrodes during cycling, Fig 6a presented the changes in VE and EE for TCF, C-TCF and Bi/C-TCF electrodes after 1000 charge-discharge cycles. For the VRFB with TCF electrode, a significant decrease in EE was observed after 300 cycles, with substantial degradation occurring after 515 cycles. This degradation was attributed to electrolyte infiltration into the carbon fibers, which substantially reduced the number of active sites on the electrode surface, leading to a pronounced decrease in both VE and EE. The C-TCF electrodes exhibited intermediate stability, showing marked VE and EE attenuation at the 816th cycle, ultimately suffering 8.57% VE loss and 9.61% EE degradation after 1000 cycles. In stark contrast, the Bi/C-TCF electrode demonstrated exceptional stability, with only a minimal reduction of 2.3% in VE and 2.2% in EE after 1000 cycles. The superior chemical stability and corrosion resistance of the Bi/C-TCF electrodes result in a longer lifespan in VRFBs. Supporting this conclusion, Fig 6b shows photographs of the electrodes after cycling. The TCF electrode, pictured on the left after 515 cycles, exhibited visible signs of corrosion, resulting in increased hardness and reduced flexibility. Conversely, the Bi/C-TCF electrode, depicted on the right, retained high flexibility even after extensive cycling. Fig 6c and 6d present SEM images of the electrodes after cycling. The SEM images of the TCF electrode revealed extensive etching on the surface, forming a sponge-like structure that indicated a loss of catalytic activity. In contrast, the surface morphology of the Bi/C-TCF electrode after 1000 cycles remained nearly identical to its uncycled state, as depicted in Fig 2d, with Bi/C nanoparticles still intact. This suggests that the Bi/C nanoparticles not only enhance the number of catalytic active sites but also serve as a protective layer for the carbon fiber substrate to prevent electrolyte infiltration. Fig 6e displays CV curves after cycling. Structural alterations in the electrode materials, including the loss of active material or reduction in catalytic sites due to prolonged cycling, resulted in a decrease in peak current densities for both TCF and Bi/C-TCF electrodes. However, the Bi/C-TCF electrode demonstrated only a 3.0% reduction in $I_{pa}$ and a 10.3% reduction in $I_{pc}$ peak currents, significantly lower than the 10.5% and 11.8% reductions observed for the TCF electrode. Furthermore, the $\Delta E_{p}$ for the Bi/C-TCF electrode decreased noticeably after cycling, while it increased for the TCF electrode, emphasizing the superior electrochemical stability of Bi/C-TCF as a negative electrode material. Detailed quantitative data are provided in Fig 6f.

## Conclusion

In this study, we successfully fabricated Bi/C-TCF electrodes for VRFBs and comprehensively evaluated their electrochemical performance and stability. The Bi/C-TCF electrodes exhibited superior electrochemical activity, as evidenced by higher VE and EE across various current densities compared to TCF electrodes. At a high current density of 300 mA/cm^2^, Bi/C-TCF electrodes maintained stable efficiencies of approximately 70%, significantly outperforming TCF electrodes. The GITT and CV results demonstrated reduced polarization and enhanced reversibility, with a significantly higher reaction rate constant K^0^(2.37 × 10^−2^ cm/s and 2.75 × 10^−2^ cm/s), attributed to the improved electron transfer kinetics and higher density of active sites provided by the Bi/C composite material. Long-term stability tests over 1000 charge-discharge cycles further highlighted the remarkable durability of Bi/C-TCF electrodes, with minimal decreases in VE and EE, which in stark contrast to the significant degradation observed in TCF electrodes. SEM conducted before and after cycling confirmed that the Bi/C particles effectively protected the carbon fiber substrate from electrolyte infiltration and maintained the structural integrity of the electrode, ensuring sustained catalytic performance. These findings indicate that Bi/C-TCF electrodes not only enhance the electrochemical activity and efficiency of VRFBs but also offer robust long-term stability, positioning them as a promising candidate for large-scale energy storage applications. The successful integration of Bi/C particles highlights the potential of carbon-based modifications in advancing the performance and reliability of VRFB electrodes, paving the way for more efficient and durable energy storage systems.

## Supporting information

S1 FigCV curves of the V^3+^/V^2+^ pair on different electrodes at varying scan rates.(a) TCF and (b) Bi/C-TCF. The fitting lines of $ln|I_{pa}|$ and |Ep−E0| for (c) V^2+^ oxidation process and (d) V^3+^ reduction process on different electrodes.(TIF)

S2 Fig (a) CV curves of C-TCF samples prepared from asphalt treated at 700°C, 800°C, and 900°C. (b) CV curves of Bi/C-TCF with Bi to C mass ratios of 1:1, 1.5:1, and 2:1.
(TIF)

S1 Table The parameters of the V^3+^/V^2+^ redox peaks on TCF at different scan rates. (DOCX)

S2 Table The parameters of the V^3+^/V^2+^ redox peaks on Bi/C-TCF at different scan rates. (DOCX)

S3 Table The fitting results of kinetic parameters. The intercepts and rate constants of the V^2+^ oxidation process and V^3+^ reduction process on TCF and Bi/C-TCF.
(DOCX)
