# Supplementary figures and images for: Carbon felt modified with bismuth and asphalt-derived carbon as a high-performance electrode for vanadium redox flow batteries
Source: PLoS One. 2025 May 28;20(5):e0324878. doi: 10.1371/journal.pone.0324878 (PMC12118884; doi:10.1371/journal.pone.0324878)

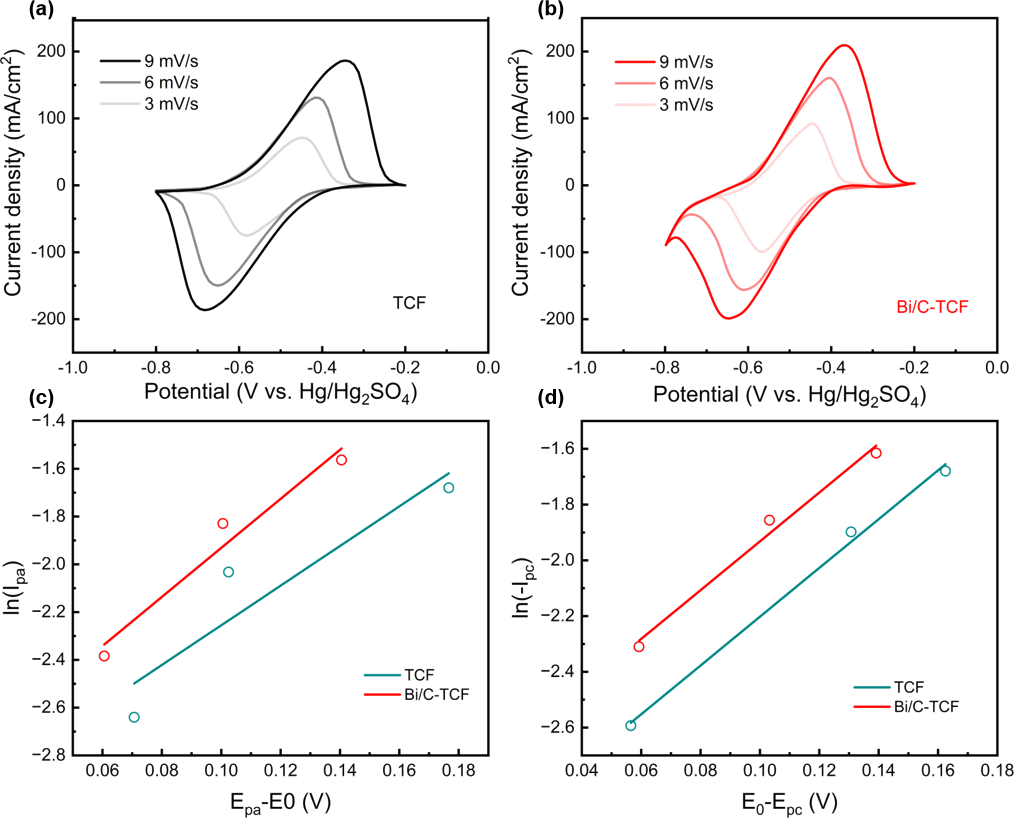

Supplement: S1 Fig — (a) TCF and (b) Bi/C-TCF. The fitting lines of ln|Ipa| and |Ep−E0| for (c) V2+ oxidation process and (d) V3+ reduction process on different electrodes. (TIF) [file pone.0324878.s001.tif]

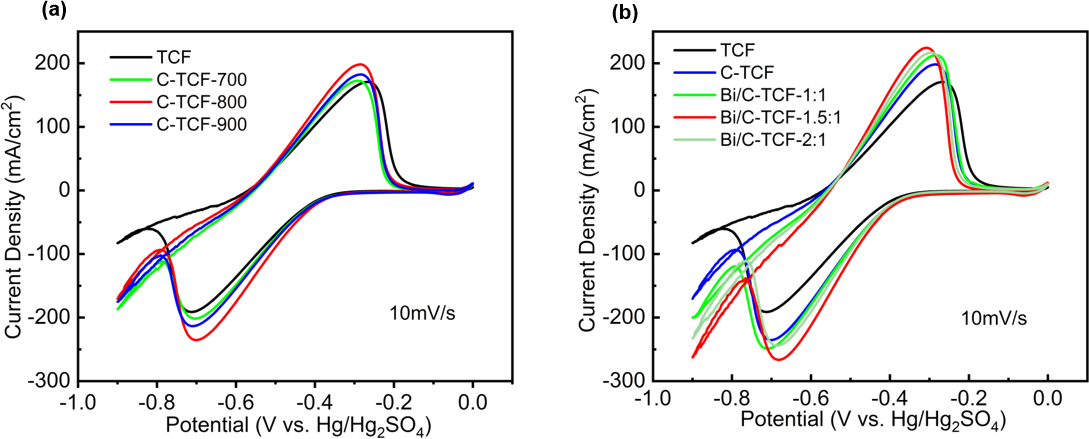

Supplement: S2 Fig — (b) CV curves of Bi/C-TCF with Bi to C mass ratios of 1:1, 1.5:1, and 2:1. (TIF) [file pone.0324878.s002.tif]
