# Supplementary material for: Carbon felt modified with bismuth and asphalt-derived carbon as a high-performance electrode for vanadium redox flow batteries
Source: PLoS One. 2025 May 28;20(5):e0324878. doi: 10.1371/journal.pone.0324878 (PMC12118884; doi:10.1371/journal.pone.0324878)
Supplement: S1 Table — (DOCX) [file pone.0324878.s003.docx]

**S1 Table .** The parameters of the V^3+^/V^2+^ redox peaks on TCF at different scan rates.

| Scan Rate | E_pa_ (V) | E_pc_ (V) | I_pa_ (mA/cm^2^) | -I_pc_ (mA/cm^2^) | | ΔE (V) | |
| --- | --- | --- | --- | --- | --- | --- | --- |
| 3 mV/s | -0.45 | -0.58 | 71.34 | 74.78 | 0.13 | |  |
| 6 mV/s | -0.42 | -0.65 | 131.02 | 149.83 | 0.23 | |  |
| 9 mV/s | -0.34 | -0.68 | 186.42 | 186.43 | 0.34 | |  |
