# Supplementary material for: Carbon felt modified with bismuth and asphalt-derived carbon as a high-performance electrode for vanadium redox flow batteries
Source: PLoS One. 2025 May 28;20(5):e0324878. doi: 10.1371/journal.pone.0324878 (PMC12118884; doi:10.1371/journal.pone.0324878)
Supplement: S3 Table — The intercepts and rate constants of the V2+ oxidation process and V3+ reduction process on TCF and Bi/C-TCF. (DOCX) [file pone.0324878.s005.docx]

**S3 Table .** The fitting results of kinetic parameters. The intercepts and rate constants of the V^2+^ oxidation process and V^3+^ reduction process on TCF and Bi/C-TCF.

| Electrode | Intercept  (anodic) | K_a_^0^ (cm/s) | Intercept  (cathodic) | K_c_^0^ (cm/s) |
| --- | --- | --- | --- | --- |
| TCF | -3.09 | 2.08×10^-2^ | -3.08 | 2.10×10^-2^ |
| Bi/C-TCF | -2.96 | 2.37×10^-2^ | -2.81 | 2.75×10^-2^ |
